# Improving accuracy of total knee component cementation: description of a simple technique

**DOI:** 10.1186/1749-799X-4-38

**Published:** 2009-10-09

**Authors:** William B Lutes, Michael A Flierl, Michael R Dayton, Steven J Morgan

**Affiliations:** 1 Department of Orthopaedic Surgery, Denver Health Medical Center, University of Colorado School of Medicine, 777 Bannock Street, Denver, CO 80204, USA

## Abstract

**Background:**

Total knee arthroplasty represents a common orthopedic surgical procedure. Achieving proper alignment of its components with the predrilled patellar and tibial peg holes prior to polymerization of the bone cement can be challenging.

**Technique:**

After establishing the femoral, patellar and tibial bone cuts, the cancellous bone around the tibial keel, as well as the peg holes for the patella and femoral components are marked with methylene blue using a cotton swab stick. If bone cement is then placed onto the cut and marked bone edges, the methylene blue leaches through the bone cement and clearly outlines the tibial keel and predrilled femoral and patellar peg holes. This allows excellent visualization of the bone preparations for each component, ensuring safe and prompt positioning of TKA components while minimizing intraoperative difficulties with component alignment while the cement hardens.

**Conclusion:**

The presented technical note helps to improve the accuracy and ease of insertion when the components of total knee arthroplasty are impacted to their final position.

## Background

The prevalence of degenerative joint disease has seen a considerable increase due to general aging of the population [1-3]. Total knee arthroplasty (TKA) represents a safe and efficacious treatment option for severe arthritis of the knee [4,5]. The volume of implanted TKA is expected to increase by 40% over the next three decades [6]. Thus, the degenerated knee has been termed "the joint of the decade" [7]. A favorable outcome of TKA depends on the optimal positioning of the components and soft tissue balancing rather than the choice of implant [8,9]. Malalignment of TKA components has been associated with knee pain [10], poor patellar tracking [11], flexion gap instability [12], loss of motion, and early implant failure [13-15]. Of note, increased prosthetic malalignment has been noted following minimally invasive total knee arthroplasty due to decreased visualization of the operative field [16,17]. Proper implant positioning and alignment during cemented TKA can be a challenging task. Malalignment of the pegs of the patella with acrylic bone cements, such as poly methylmethacrylate (PMMA), prior to cementation requires rotation of the patella, which may result in significant yet unwarranted extrusion of PMMA. Impaction of the tibial component in improper rotational alignment may create a larger space for the keel. These seemingly small errors shorten valuable working time with the PMMA and could become catastrophic if the polymerization phase begins and the prosthesis is in improper alignment.

In the present technical note, we describe a simple modification prior to cementation of TKA components to ensure alignment of patella peg holes, tibial keel, and femoral prosthesis. This surgical technique can be performed in as little as 30 seconds. It thus marginally prolongs the surgical case while helping to avoid intraoperatively repositioning maneuvers of implant components.

## Surgical technique

**Figure 1:**
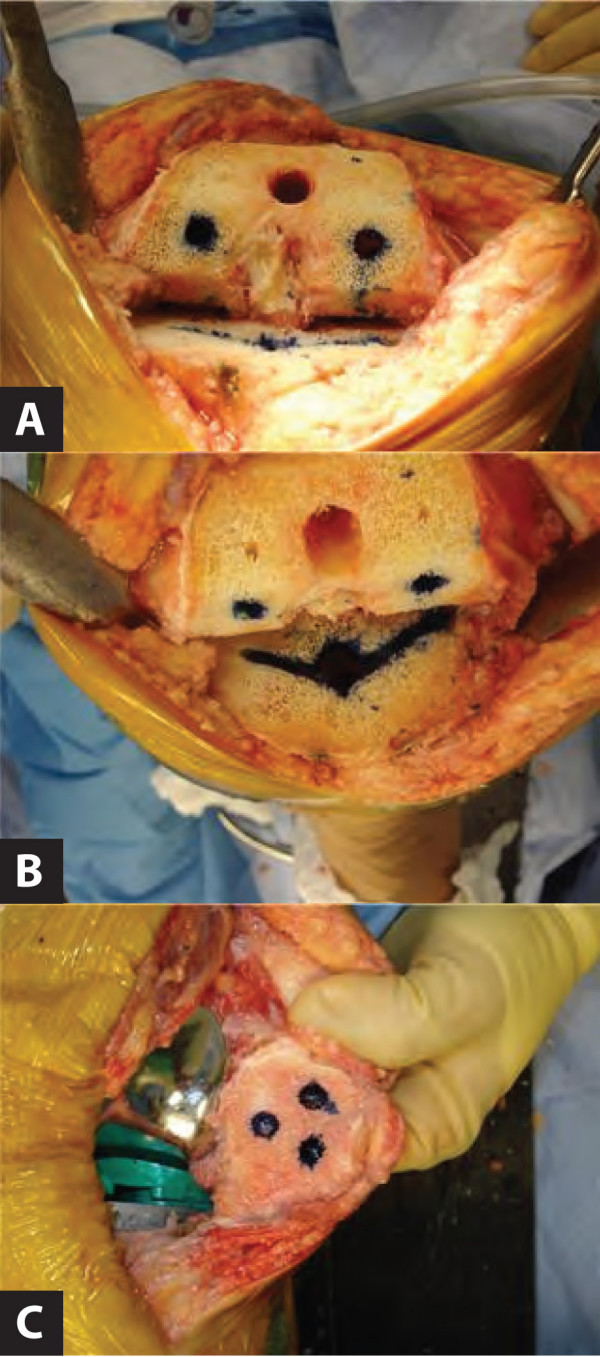
**Intraoperative images of the femur (A), tibia (B) and patella (C) prepared with methylene blue prior to cementation**.

The standard TKA technique is pursued according to the surgeon's preference. Once the femoral, patellar and tibial bone cuts are established and the knee is balanced correctly, the cut bone surfaces are prepared for cementation in the choosen standard fashion. At this point, the cancellous bone around the tibial keel, as well as the peg holes for the patella and femoral components are marked with methylene blue using a cotton swab stick. Figure 1 depicts the cut bony surfaces following intraoperative staining of the peg holes and the tibial keel. Bone cement is then placed onto the cut bone edges and manual digital pressure is applied to the cement so the bone cuts become evident. The methylene blue leaches through the bone cement and clearly outlines the tibial keel and predrilled femoral and patellar peg holes.

**Figure 2:**
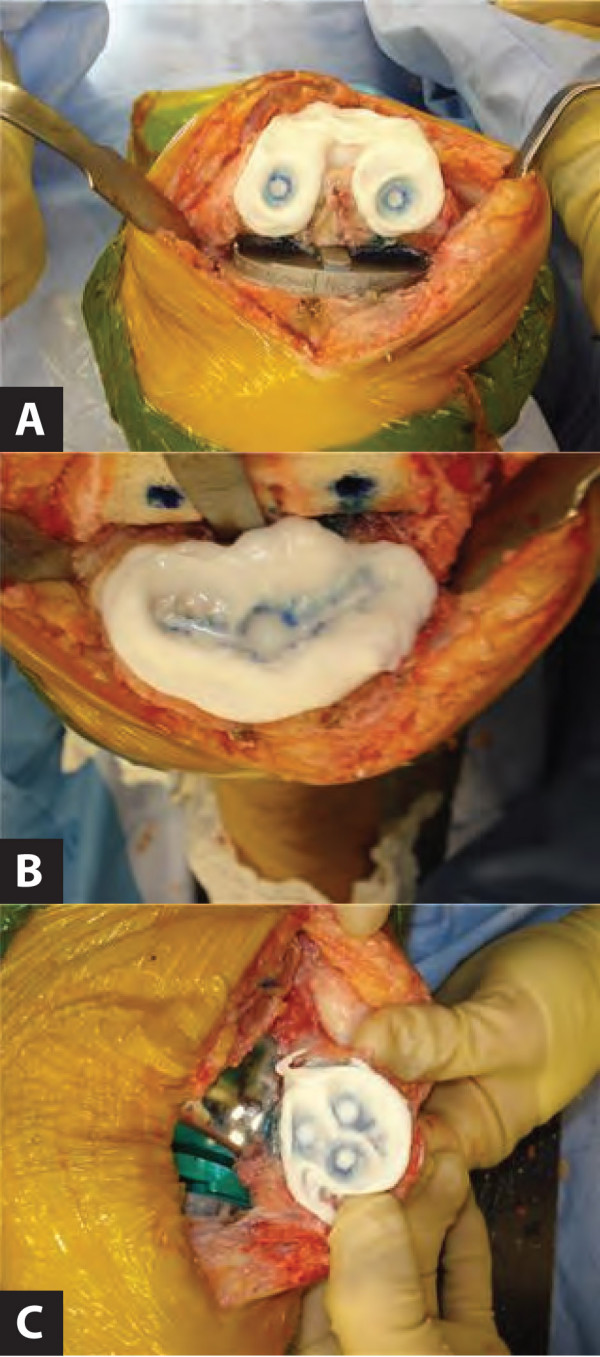
***In situ *illustration of the femur (A), tibia (B) and patella (C) with polymethylmethacrylate applied**. Note the obvious leakage of the methylene blue through the bone cement, clearly outlining the tibial keel and predrilled femoral and patellar peg holes.

The presented technique allows for excellent visualization for the appropriate placement of each component in its prepared location (Figure 2). Intraoperative delineation of the tibial keel and the predrilled pegholes thus ensures safe and prompt positioning of TKA components and avoids unwarranted intraoperative struggle with component placement while the cement hardens. As the described technique adds only about 30 seconds, minimal additional time for bone-cement preparation is required. Having the appropriate positioning marked prior to component impaction or application of the patella clamp allows for fast completion of the surgical procedure avoiding repositioning of any components.

## Discussion

Total knee arthroplasty is a frequently performed surgical procedure [7]. However, it is combined with inherent risks of misalignment of implant components, which is likely to result in poor clinical and long-term outcome. In the present report, we describe a simple and straight forward technical trick that helps to insure appropriate intraoperative alignment of the TKA components. The methylene blue method assists in creating reproducibly good results during component impaction and has been successfully used in over 1000 cases at our institution. It represents a safe and efficacious method that adds only about 30 seconds to the standard TKA procedure.

However, as the alignment of each component is highly depended on the location of predrilled peg holes, the presented technique can only allow better visualization for component implantation. As a result, rotational accuracy and alignment cannot be improved when predrilled peg holes are rotationally malaligned. In addition, surgeons routinely applying the cement to the prosthesis prior to component placement will not benefit from the described technical trick.

This small change in technique prior and during cementation may prevent intraoperative complications and struggles with optimizing the implant alignment during the 6-10 minute time-window until the PMMA cures [18], and thereby help avoide unwarranted intraoperative complications and maximize patient safety. We hope that our practical note may facilitate and assist other surgeons performing TKAs on a routine basis.

## Competing interests

The authors declare that they have no competing interests.

## Authors' contributions

WBL, MRD and SJM designed the manuscript. MAF and SJM wrote the manuscript. All authors approved the final version of this review.
